# Editorial

**Published:** 1891-05

**Authors:** 


### Editorial.

### PRACTICAL HINTS.

Now that the Summer is approaching, and in feeling seems actually upon us, it behooves all to see that their systemic condition is in perfect trim, in order that they may be less susceptible to the complaints which are likely to occur at this season. Especially does the above apply to the physician whose work is a song of daily and nightly toil. "Spring fever," though often synonymous with laziness, is frequently more than an "idle fancy." The warm weather brings a laxness of feeling which is participated in by all the various organs of the body. Cold weather demanded an increased supply of food and muscular exertion that the bodily temperature and conditions so necessary for its well being might be maintained. As the season advances this demand grows less and bodily exertion reaches its minimum. The system becomes torpid, as it were. The various organs of the body not being called upon for the same amount of work, grow languid, and so the various secretions and excretions are not up to their normal standard. The various bodily functions must be attended to. The liver, with its many imperfect functions, is especially liable to take on this condition of inactivity. Its biliary function is frequently deranged. The skin looks sallow, the tongue coated, a feeling in the morning as if one's night's rest had not been refreshing. All these, with other feelings of lassitude, tell us that we are not in "working trim." To these must be added the susceptibility to colds. If there is any one thing that is harrassing, it is a "summer cold." One cannot be too careful about changing their flannels. Do it gradually, or wait until you know the season has fully settled. Keep the bowels open, if only with a glassful of cold water in the morning before breakfast. Small doses of calomel will prove exceedingly efficacious, both as a tonic and laxative. Cool bathing daily promotes the functions of our nervous system and adds a stimulus to the proper activity of our various bodily organs. Take a sponge bath every morning. Don't over eat, but sustain the body with nutritious diet. Fruit is always beneficial. Finally, let the physician see that he is well protected from the chilling air of night, when his peaceful slumbers are disturbed by some nocturnal visitor.

### FREQUENCY OF DIABETES.

A very valuable and thorough treatise on Diabetes has lately appeared from the pen of Dr. Purdy, of Queens College. The author shows a thorough familiarity with the subject, and gives some important facts from a statistical point of view. In his historical sketch of the disease he gives some important facts in regard to its prevalence in the United States. He deprecates the fact that our country has no bureau of vital statistics, differing as it does from all other civilized countries; but his data, as given, were gathered from the last census.

In estimating the frequency of death from diabetes in the various States, he estimates the number per 1,000. Vermont stands first, with a rate of 6.36, with Maine second, her rate being 4.41. The rate per 1,000 for Georgia is 1.11. The author, from a comparison of the various States, deduces the fact that the difference in the death rate is determined by the altitude of the country. The higher it is, the greater increase of the death rate.

Another remarkable fact is given that in the mortality reports of the United States for 1880 they furnish not a single death from diabetes in either the Indian or Chinese population of the country. He seems to think it a race peculiarity—probably a failing to report.

The author states that from a comparison of the records, the relative mortality has been decidedly on the increase for the last forty years. He says that between 1860 and 1870 the death rate increased enormously, and attributes it to the fact that with the war of 1860 came inflation of currency and hitherto unknown abundance of money. One can readily see that the South was entirely foreign to the author's mind when he made the above statement. We should rejoice that though the war brought poverty to the Southerners, it precluded diabetes from our midst.

### CONGRESS OF AMERICAN PHYSICIANS AND SURGEONS.

The meetings of the Congress of American Physicians and Surgeons will be held in Washington from 3 to 6 p. m., September 22d, 23d, 24th and 25th, 1891. William Pepper, Chairman of the Executive Committee.

### RULES FOR BATHING.

Now, as the season for open-air bathing is about to open, the following rules, as culled from the *Lactopeptine Medical Annual*, will be of interest and benefit, if complied with.

Avoid bathing within two hours after a meal. Avoid bathing when exhausted by fatigue or from any other cause.

Avoid bathing when the body is cooling after perspiration. Avoid bathing altogether in the open air, if after having been a short time in the water, it causes a sense of chilliness and numbness of the hands and feet.

Bathe when the body is warm, provided no time is lost in getting into the water.

Avoid chilling the body by sitting or standing undressed on the banks or in boats, after having been in the water.

Avoid remaining too long in the water; leave the water immediately, if there is the slightest feeling of chilliness.

The vigorous and strong may bathe early in the morning, on an empty stomach. The young, and those who are weak, had better bathe two or three hours after a meal—the best time for such, is from two to three hours after breakfast.

Those who are subject to attacks of giddiness or faintness, and those who suffer from palpitation and other sense of discomfort of the heart, should not bathe without first consulting their medical adviser. W. A. C.

---

Dr. M. M. EVANS has removed from Florence to Tyro, Ark.

---

NORTH CAROLINA STATE MEDICAL SOCIETY.—The thirty-eighth annual meeting of the North Carolina State Medical Society will be held in Asheville, May 26th, 27th and 28th.

---

The *New York Polyclinic* is maintaining its usual position as the pioneer post-graduate school in the United States. The strictly enforced rule of not admitting graduates of irregular schools, and of not issuing a certificate even to a regular graduate unless he can give evidence that he is still in regular practice, has not only increased the attendance at this school, but has helped to gather in the class at the Polyclinic a better grade of students than in previous years.

